# Transethmoidal nasal meningocele and CSF fistula in a child with recurrent bacterial meningoencephalitis

**DOI:** 10.1186/1471-2334-14-S7-P74

**Published:** 2014-10-15

**Authors:** Anca Drăgănescu, Anuța Bilaşco, Angelica Vişan, Codruț Sarafoleanu, Claudiu Manea, Cornelia Dogaru, Diana Slavu, Monica Luminos

**Affiliations:** 1 National Institute for Infectious Diseases "Prof. Dr. Matei Balş", Bucharest, Romania; 2 Carol Davila University of Medicine and Pharmacy, Bucharest, Romania; 3 “Sf. Maria” Clinical Hospital, Bucharest, Romania

## Background

Recurrent bacterial meningitis in children poses a considerable diagnostic challenge due to its multiple etiologies. Making an early diagnosis is crucial in preventing further episodes that could lead to a potentially life threatening condition, neurologic sequelae and psychological trauma due to multiple invasive investigations. Recurrent bacterial meningitis has multiple underlying conditions, but it is most frequently caused by anatomic intracranial or lumbosacral defects (encephaloceles, meningocele, temporal bone malformations, skull fracture, dermoid cyst of the lumbosacral spine). Other predisposing conditions are different types of immunodeficiency (immunoglobulin deficiency, complement deficiency, HIV, asplenia) and chronic infections of the middle ear and paranasal sinuses.

## Case report

We report the case of a 6 year-old child with recurrent bacterial meningoencephalitis with a transethmoidal meningocele with corticospinal fluid fistula. The child was admitted to our intensive care unit for *Streptococcus pneumoniae* meningitis. She suffered from 4 episodes of bacterial meningitis in the previous year. The last brain computed tomography and MRI showed no signs of ear or paranasal sinus infection, but the MRI identified a slight asymmetry of the cribriform plates. The patient was vaccinated against pneumococcus and further multiple otolaryngology investigations with nasal endoscopy made a clear diagnosis of transethmoidal nasal meningocele of 2 mm diameter. An intranasal endoscopic surgical procedure was performed.

## Conclusion

Making an accurate diagnosis of a base skull malformation makes it possible to perform necessary surgical intervention and thereby to prevent further episodes of bacterial meningitis. Initial imaging investigation of recurrent bacterial meningitis should include a contrast enhanced thin section CT scan of the temporal bone and anterior skull base, including the paranasal sinuses and an MRI imaging to detect CSF leakage. In our case only repeated otolaryngology endoscopic examination could identify the meningocele, underlying the need for multiple specialty interaction in the management of recurrent meningitis.

